# Supplementary material for: Origin of dendrite-free lithium deposition in concentrated electrolytes
Source: Nat Commun. 2023 May 9;14:2655. doi: 10.1038/s41467-023-38387-8 (PMC10169806; doi:10.1038/s41467-023-38387-8)
Supplement: Supplementary file 1 — Supplementary Information [file 41467_2023_38387_MOESM1_ESM.pdf]

Supplementary Information

Origin of dendrite-free lithium deposition in concentrated electrolytes

Yawei Chen[1,6], Menghao Li[2,3,6], Yue Liu[4,6], Yulin Jie[1], Wanxia Li[1], Fanyang Huang[1], Xinpeng Li[1], Zixu He[1], Xiaodi Ren[1], Yunhua Chen[5], Xianhui Meng[5], Tao Cheng[4,*], Meng Gu[2,*], Shuhong Jiao[1,*], Ruiguo Cao[1,*]

[1]Hefei National Laboratory for Physical Science at Microscale, CAS Key Laboratory of Materials for Energy Conversion, Department of Materials Science and Engineering, University of Science and Technology of China, Hefei 230026, China.

[2]Department of Materials Science and Engineering, Southern University of Science and Technology, Shenzhen 518055, China.

[3]School of Materials Science and Engineering, Harbin Institute of Technology, Harbin 150001, China.

[4]Institute of Functional Nano and Soft Materials (FUNSOM), Soochow University, Suzhou 215123, China.

[5]NIO Incorporation, Shanghai, 201800, China.

[6]These authors contributed equally: Yawei Chen, Menghao Li, Yue Liu.

[*]Corresponding authors: tcheng@suda.edu.cn; gum@sustech.edu.cn; jiaosh@ustc.edu.cn; rgcao@ustc.edu.cn.

Supplementary Figures

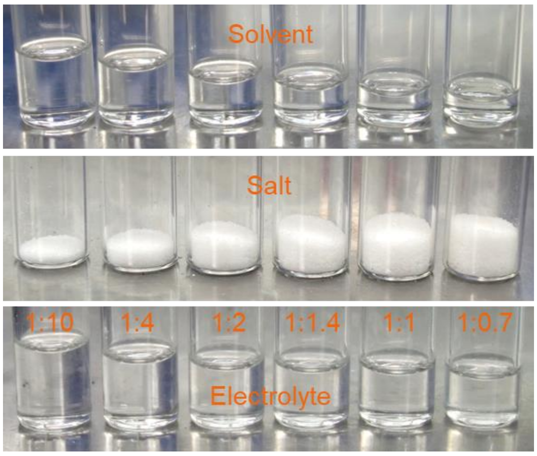

Supplementary Figure 1 | Optical images of the electrolytes. Images of LiFSI/DME solutions with various salt-to-solvent molar ratios and their corresponding amounts of solvent (DME) and salt (LiFSI).

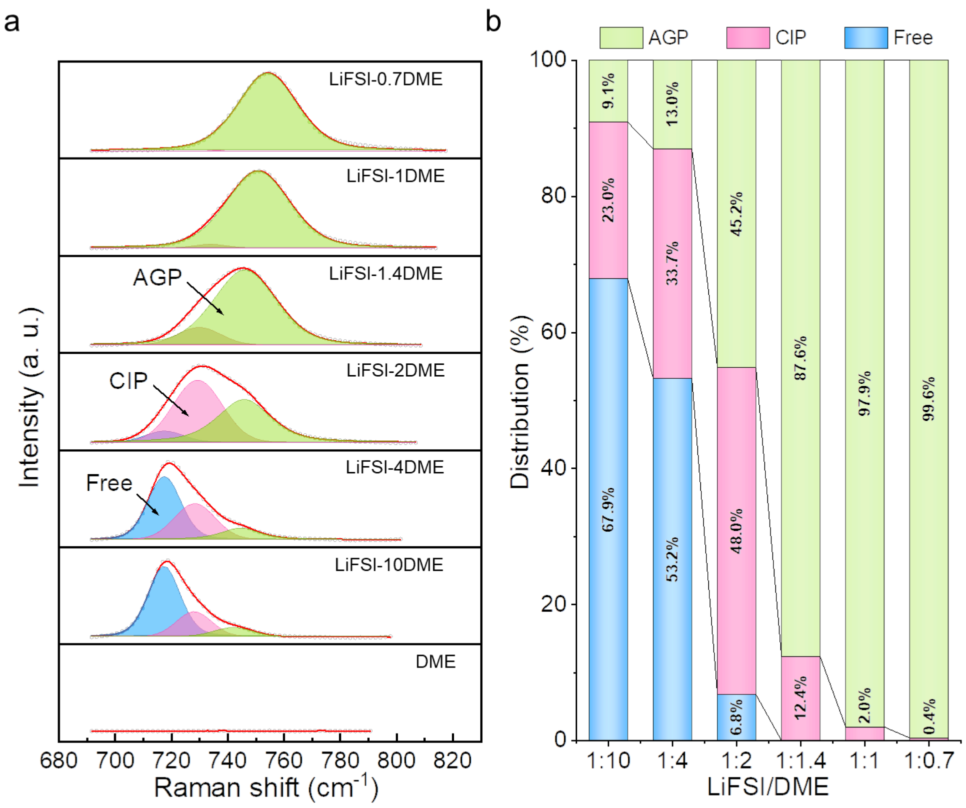

Supplementary Figure 2 | Solvation structure characterization by Raman spectroscopy. (a) Raman spectra of LiFSI/DME electrolytes with various molar ratios. (b) Fitting results of free FSI^-, CIP and AGP ratios in LiFSI/DME electrolytes.

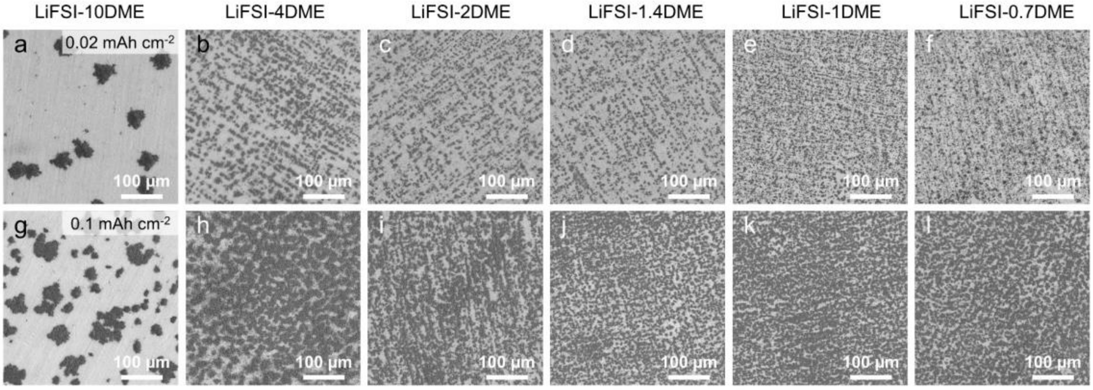

Supplementary Figure 3 | Li deposition morphology with low capacities. Low magnification SEM images of Li deposited on Cu foils with the capacities of (a-f) 0.02 mAh cm^{-2} and (g-l) 0.1 mAh cm^{-2} in LiFSI-xDME electrolytes (x=10, 4, 2, 1.4, 1, 0.7, respectively). The current density is 0.5 mA cm^{-2}.

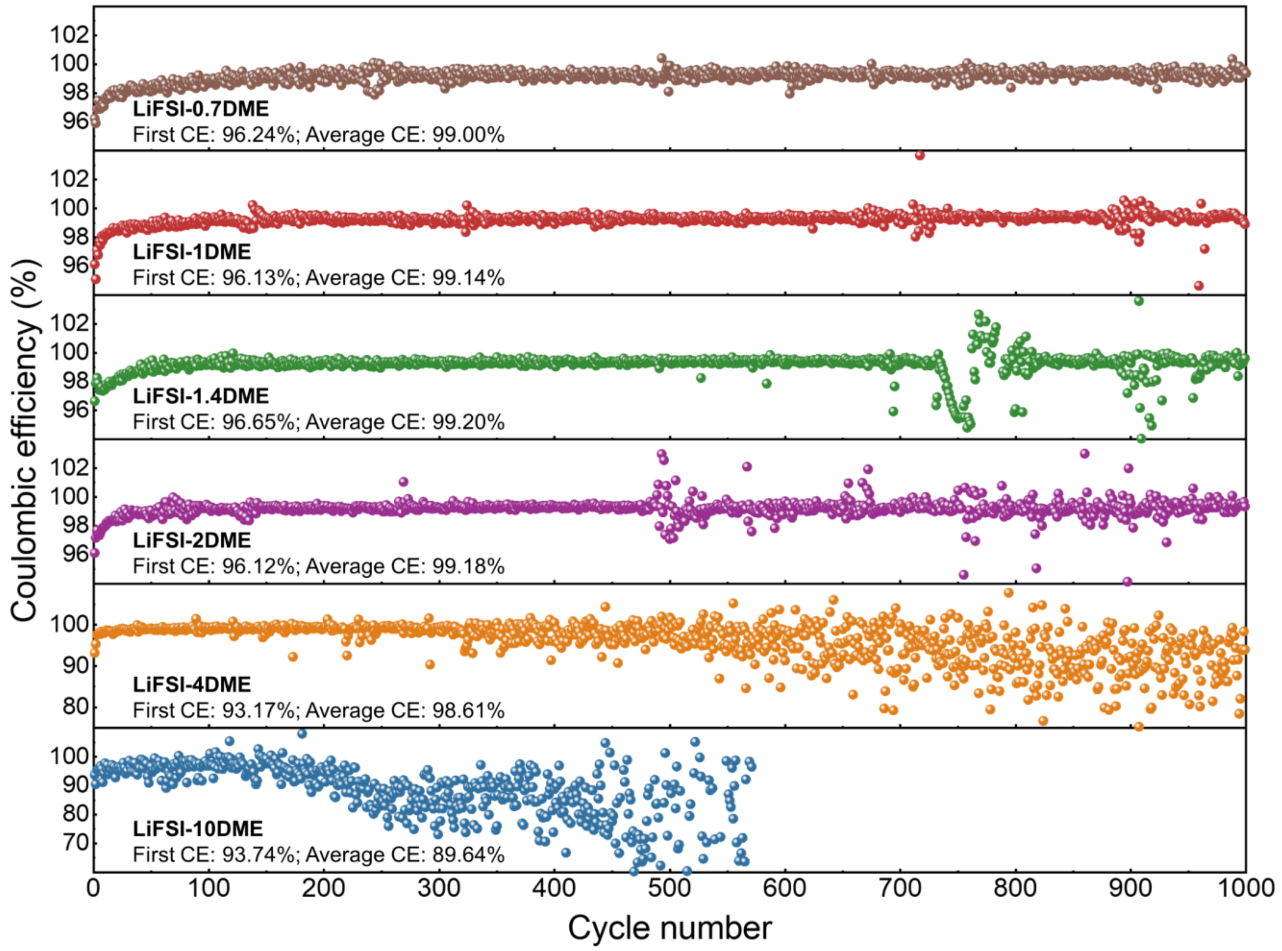

Supplementary Figure 4 | CEs in Li/Cu half cells. Long-term cycling Coulombic efficiencies of Li/Cu half cells in LiFSI-xDME electrolytes (x=10, 4, 2, 1.4, 1, 0.7, respectively). The average CEs are calculated from the first 500 cycles.

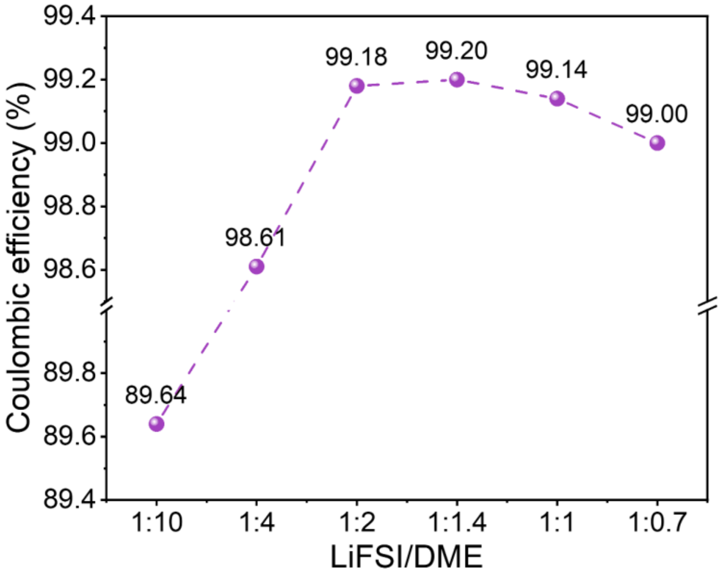

Supplementary Figure 5 | CE volcano plot. Average Li CEs of the first 500 cycles for various electrolytes.

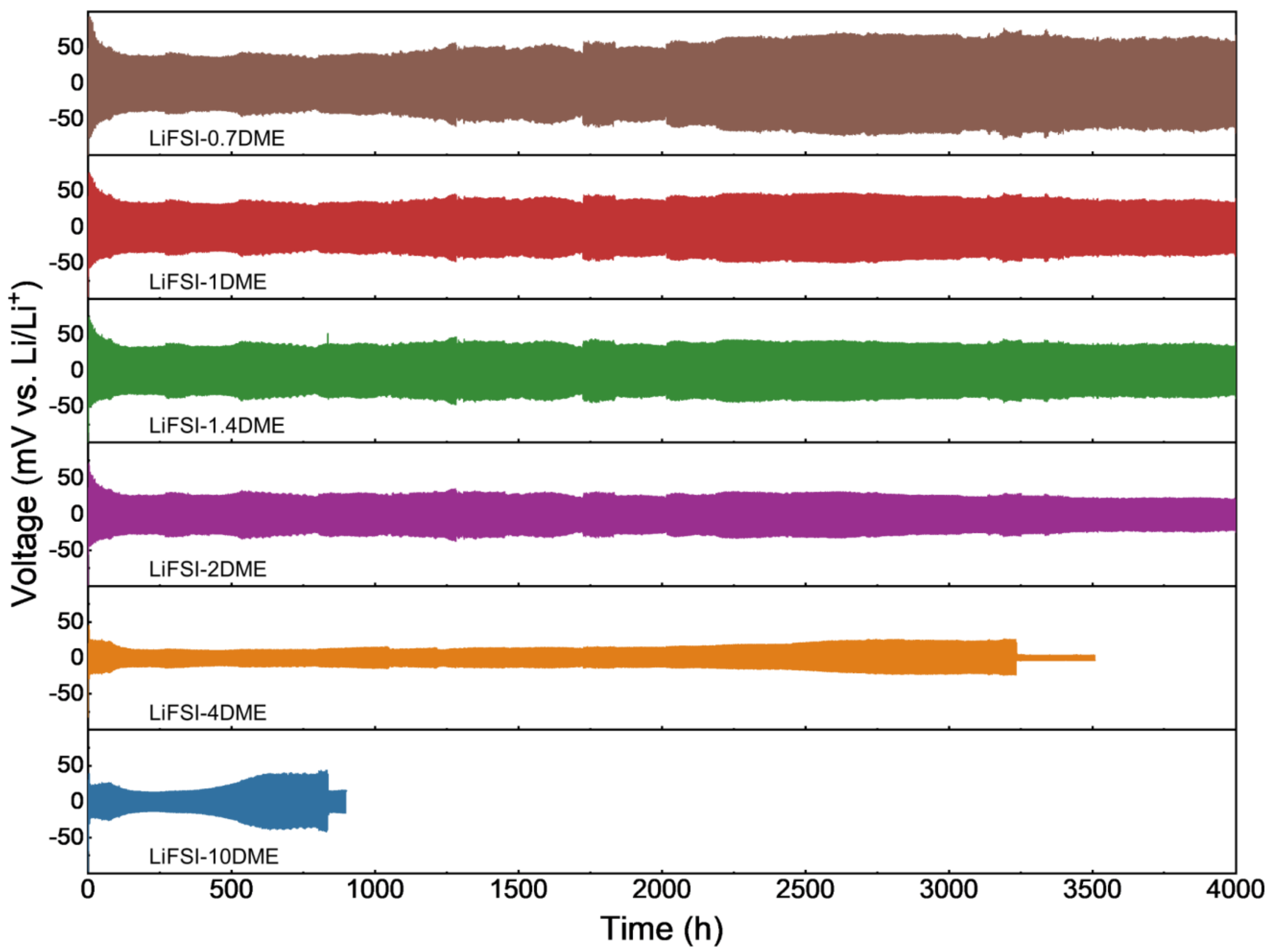

Supplementary Figure 6 | Li/Li symmetric cells. Long-term cycling performance of Li/Li symmetric cells in LiFSI-xDME electrolytes (x=10, 4, 2, 1.4, 1, 0.7, respectively).

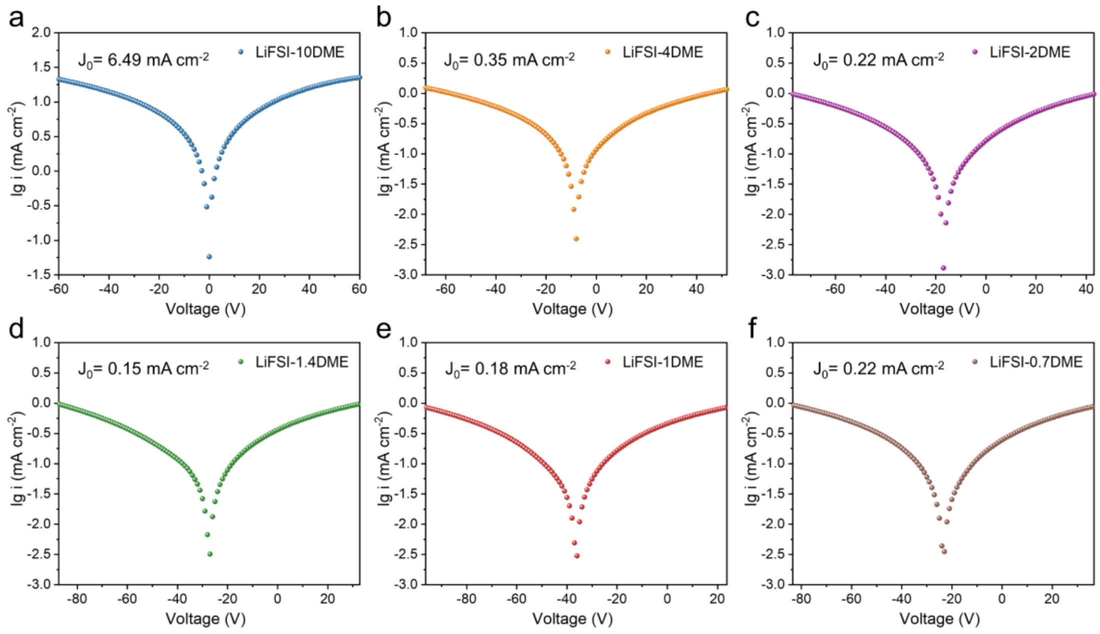

Supplementary Figure 7 | Exchange current density calculation. Tafel plots for Li plating/stripping in (a-f) LiFSI-xDME electrolytes (x=10, 4, 2, 1.4, 1, 0.7, respectively).

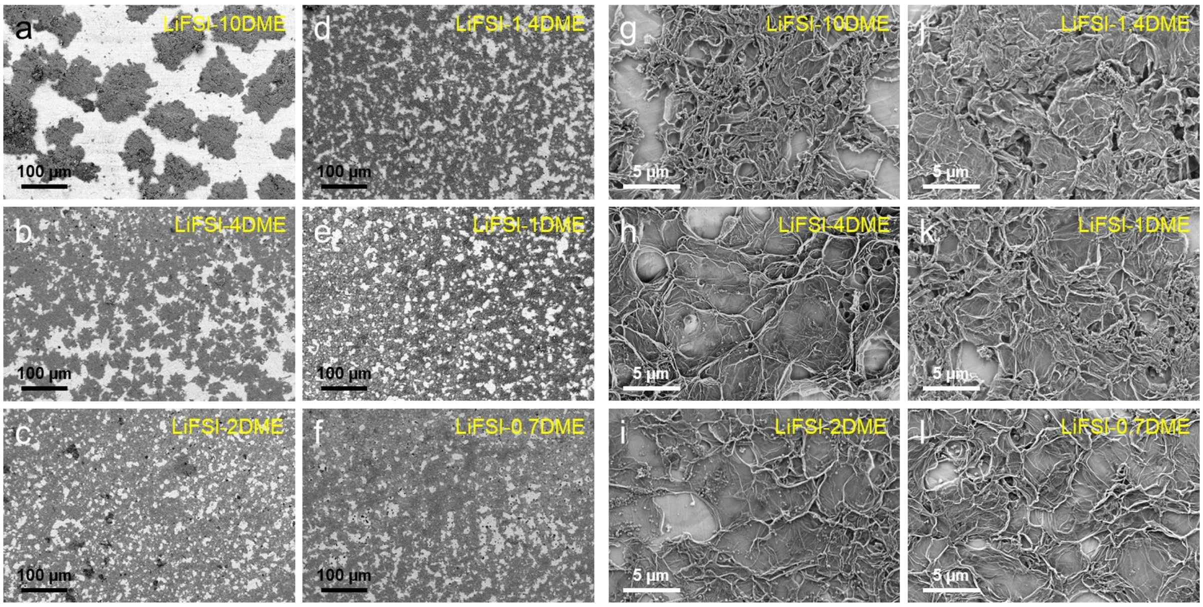

Supplementary Figure 8 | SEM images of Cu electrodes after Li stripping. SEM images of the Cu electrodes after one plating/stripping cycle (0.5 mA cm^{-2} for 1 mAh cm^{-2}) in different electrolytes: (a, g) LiFSI-10DME; (b, h) LiFSI-4DME; (c, i) LiFSI-2DME; (d, j) LiFSI-1.4DME; (e, k) LiFSI-1DME; (f, l) LiFSI-0.7DME.

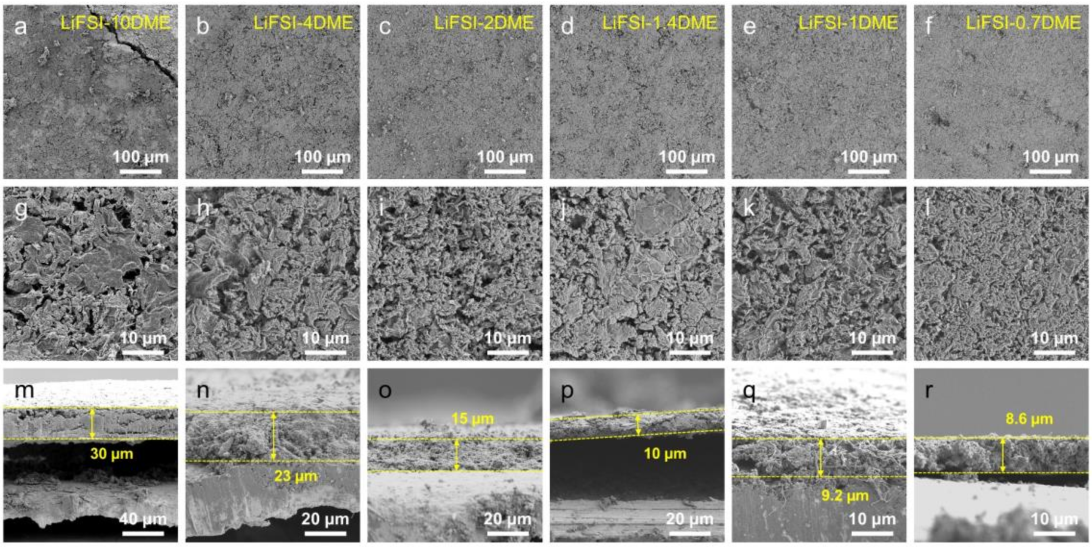

Supplementary Figure 9 | SEM images of Cu electrodes after long cycle. Top-view SEM images (a-l) and cross-sectional SEM images (m-r) of the Cu electrodes after 65 plating/stripping cycles in different electrolytes.

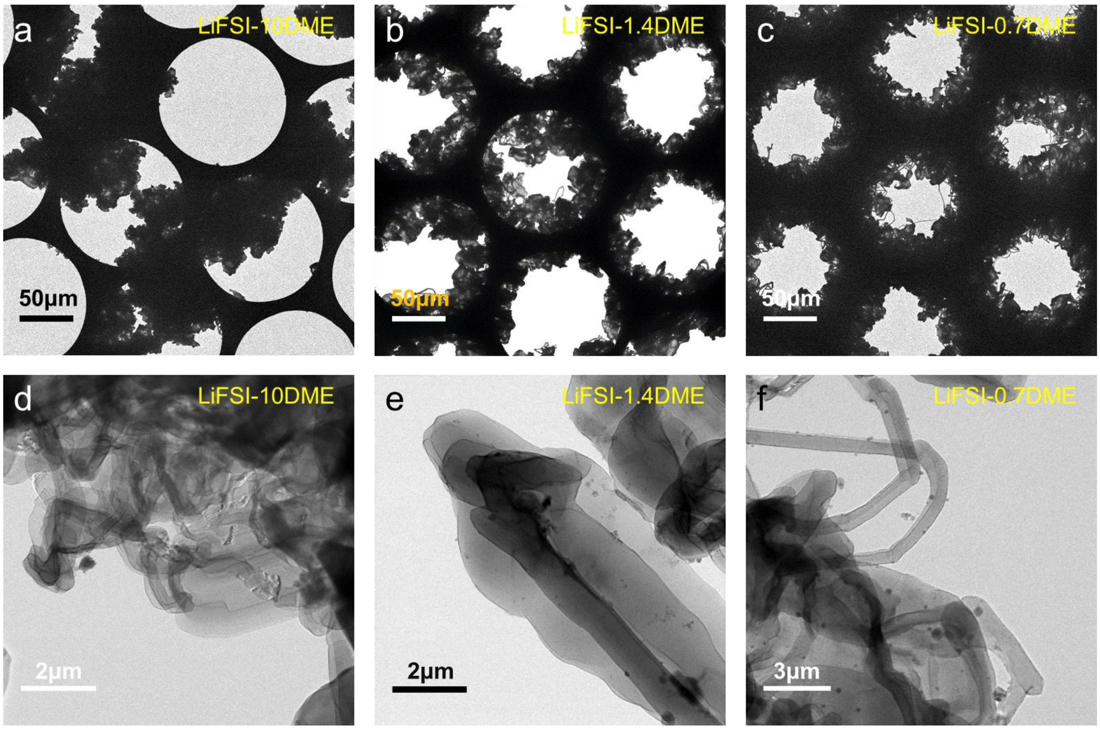

Supplementary Figure 10 | Low magnification Cryo-TEM images. Cryo-TEM images of deposited Li in different electrolytes: (a, d) LiFSI-10DME; (b, e) LiFSI-1.4DME; (c, f) LiFSI-0.7DME. The current density is 0.5 mA cm^{-2}, and the capacity is 0.5 mAh cm^{-2}.

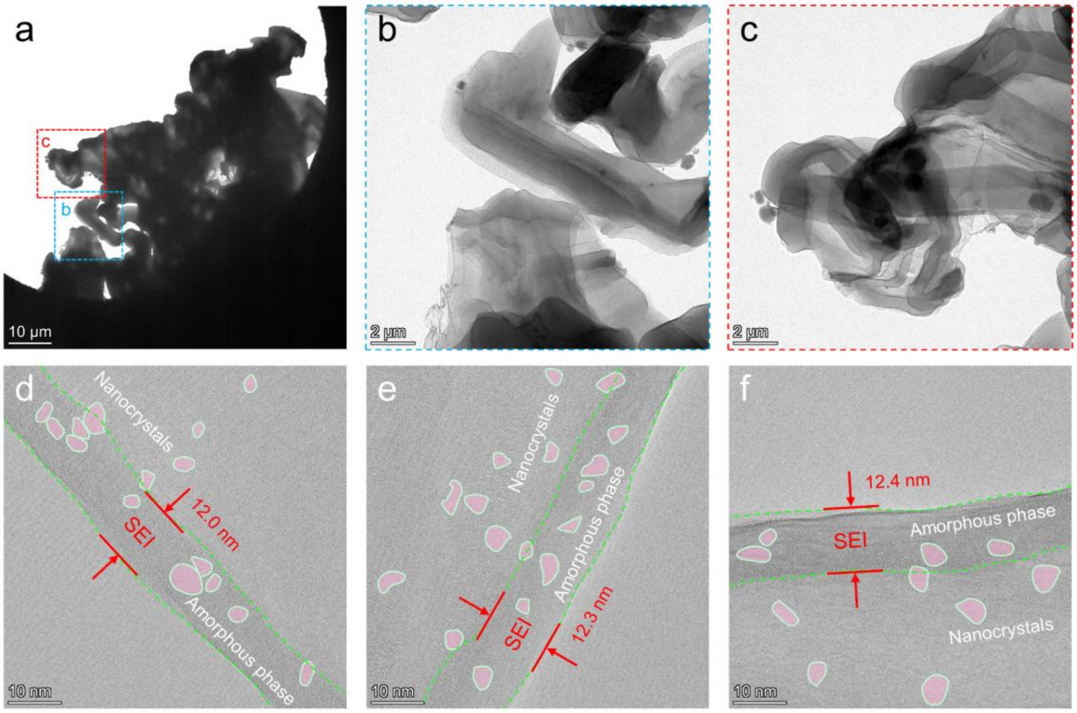

Supplementary Figure 11 | Li morphology and SEI structure in the low concentration electrolyte at a low current density. Cryo-TEM images of 0.5 mAh cm^{-2} deposited Li (a-c) and SEI (d-f) in the LiFSI-10DME electrolyte. The current density is 0.1 mA cm^{-2}.

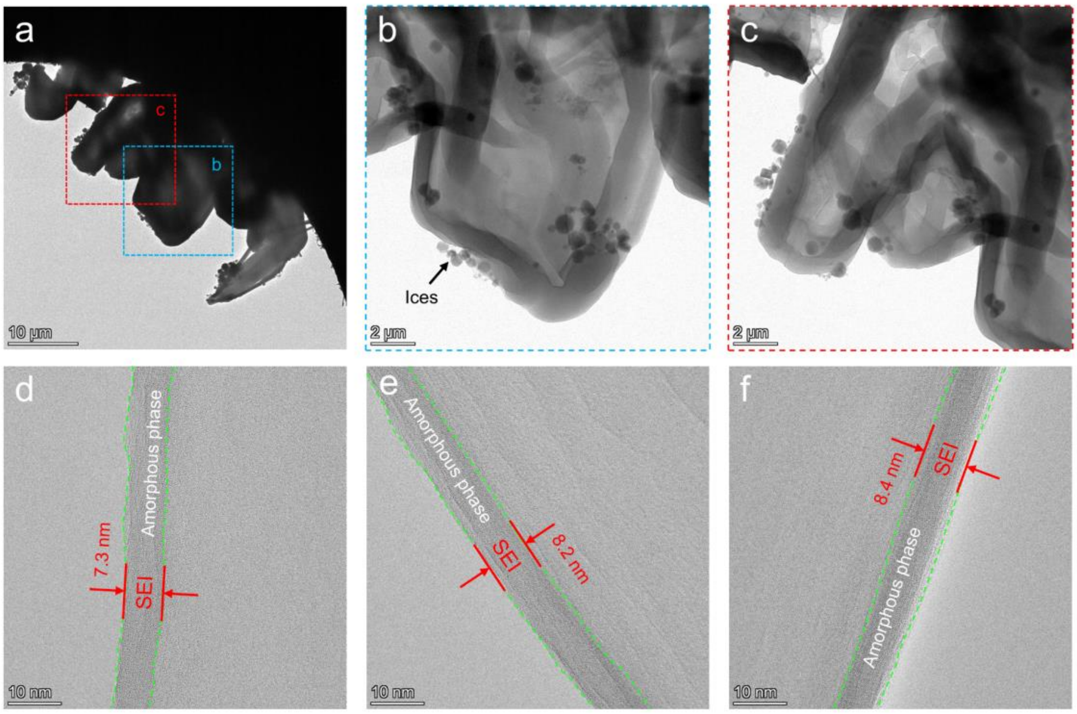

Supplementary Figure 12 | Li morphology and SEI structure in the high concentration electrolyte at a low current density. Cryo-TEM images of 0.5 mAh cm^{-2} deposited Li (a-c) and SEI (d-f) in the LiFSI-1.4DME electrolyte. The current density is 0.1 mA cm^{-2}.

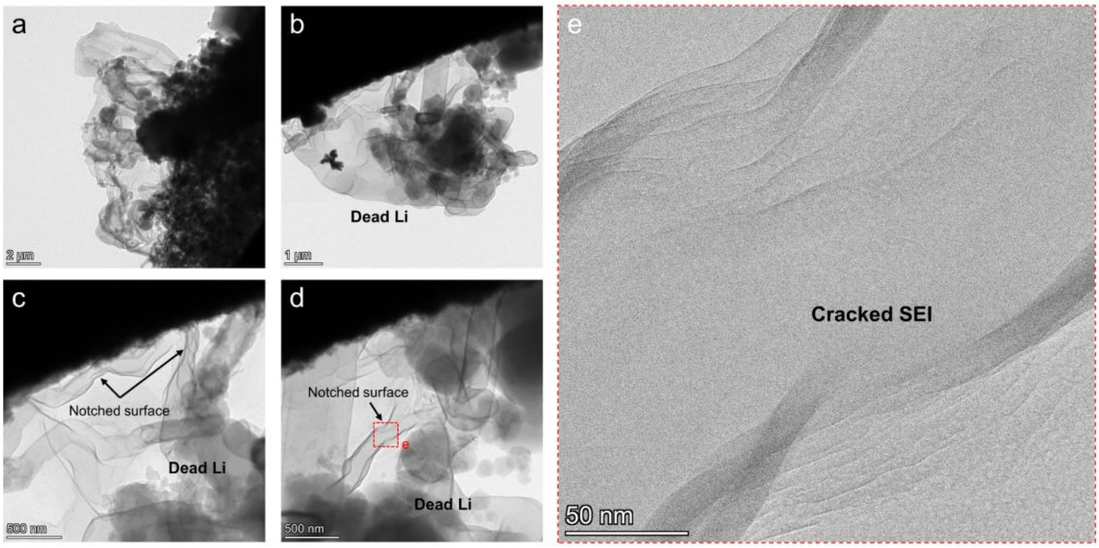

Supplementary Figure 13 | Li morphology and SEI structure after stripping in the low concentration electrolyte. Cryo-TEM images of the dead Li (a-d) and SEI (e) after stripping after 1 cycle in the LiFSI-10DME electrolyte.

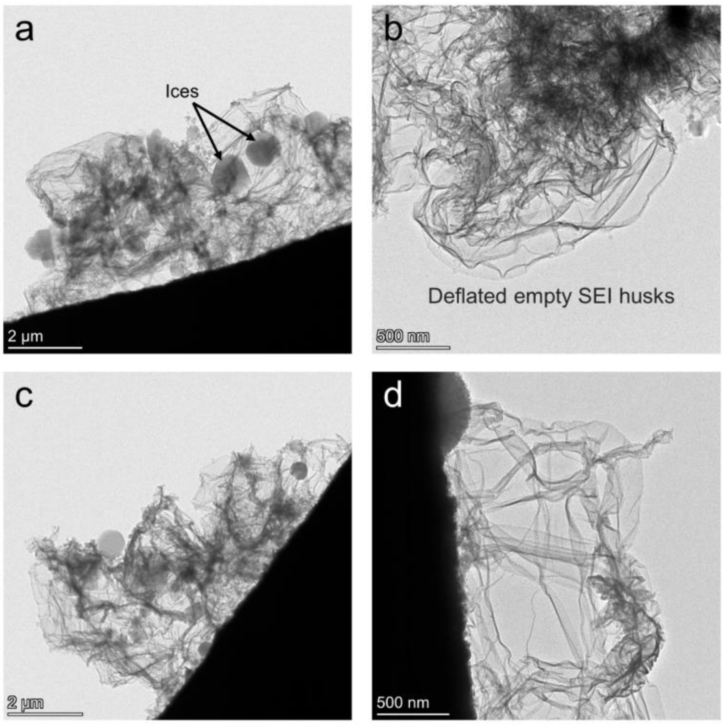

Supplementary Figure 14 | Li morphology and SEI structure after stripping in the high concentration electrolyte. (a-d) Cryo-TEM images of the SEI after stripping after 1 cycle in the LiFSI-1.4DME electrolyte.

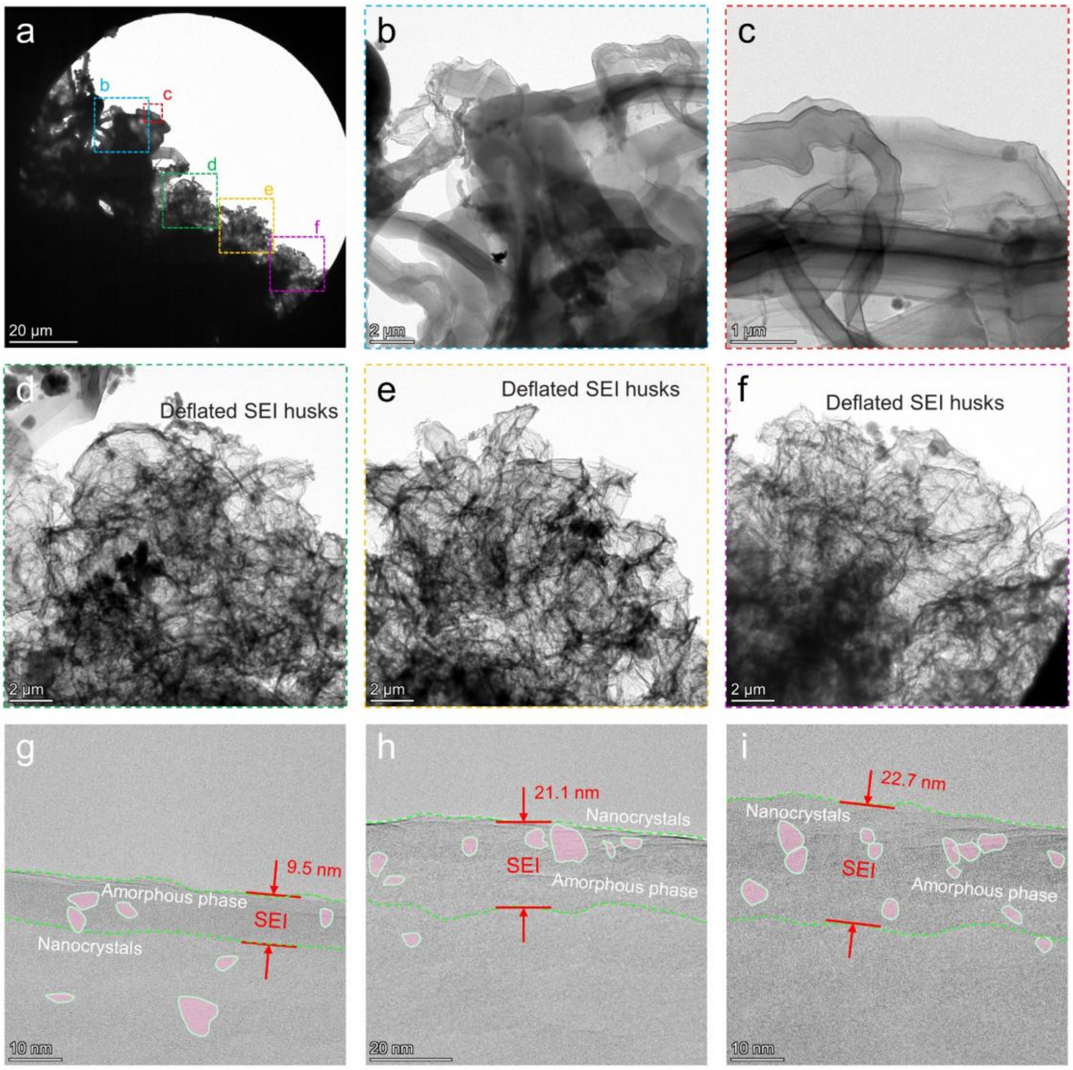

Supplementary Figure 15 | Li morphology and SEI structure after multiple cycles in the low concentration electrolyte. Cryo-TEM images of 0.5 mAh cm^{-2} deposited Li (a-c), deflated SEI husks (d-f) and SEI (g-i) after 5 cycles in the LiFSI-10DME electrolyte.

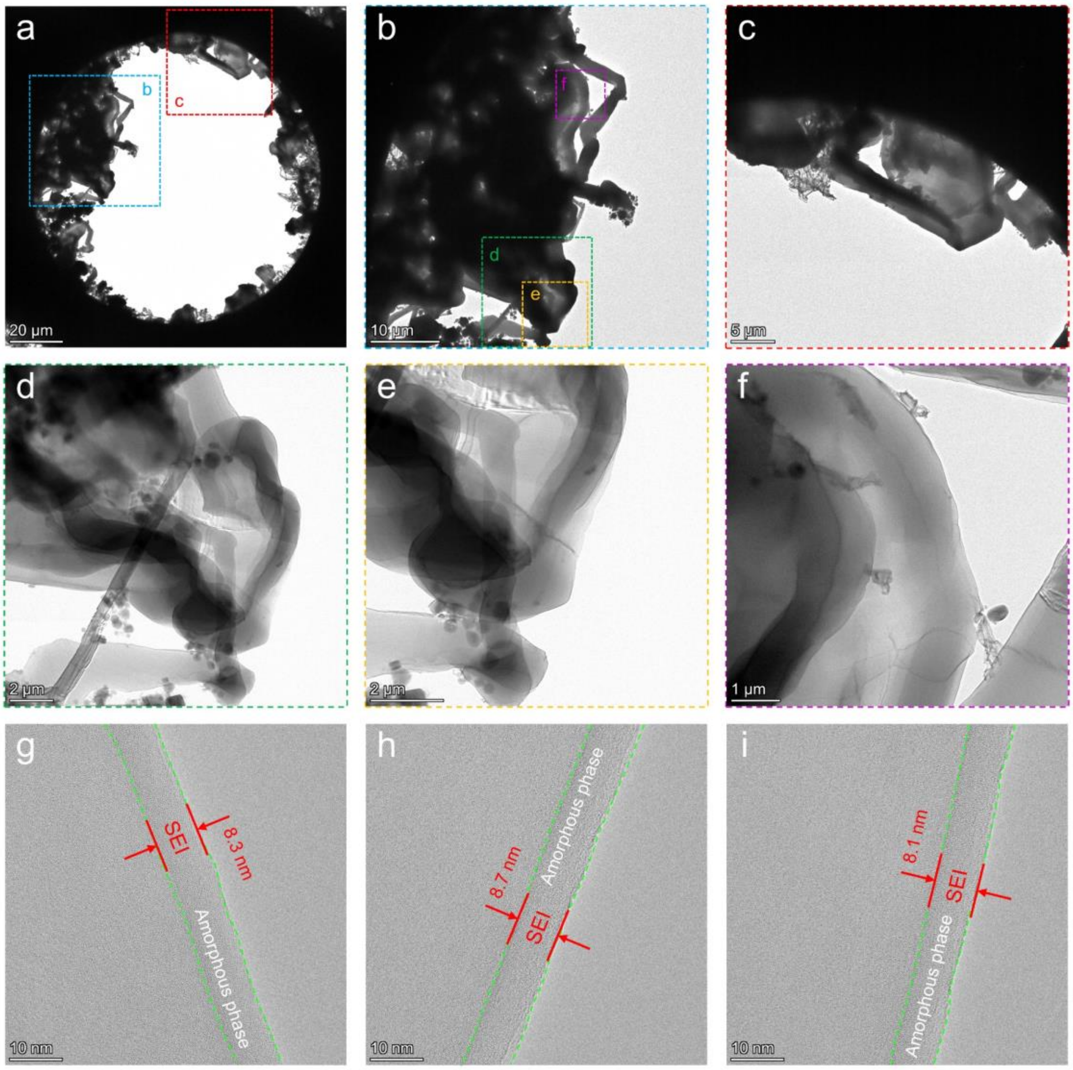

Supplementary Figure 16 | Li morphology and SEI structure after multiple cycles in the high concentration electrolyte. Cryo-TEM images of 0.5 mAh cm^{-2} deposited Li (a-f) and SEI (g-i) after 5 cycles in the LiFSI-1.4DME electrolyte.

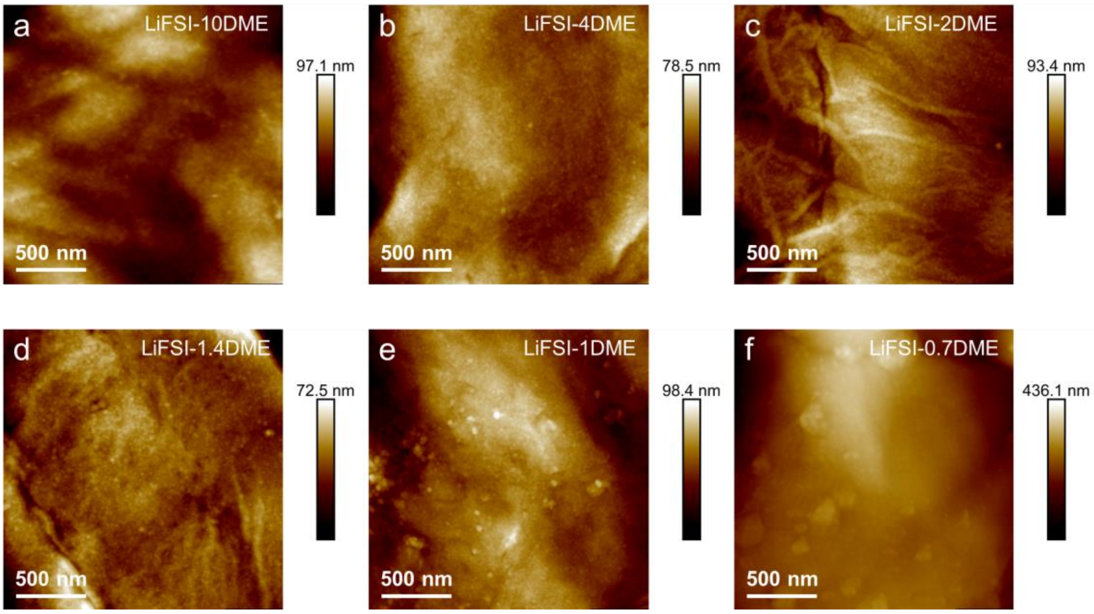

Supplementary Figure 17 | Li morphology characterization by AFM. (a-f) AFM height images of 1 mAh cm^{-2} Li deposited on Cu foils in LiFSI-xDME electrolytes (x=10, 4, 2, 1.4, 1, 0.7, respectively). The current density is 0.5 mA cm^{-2}.

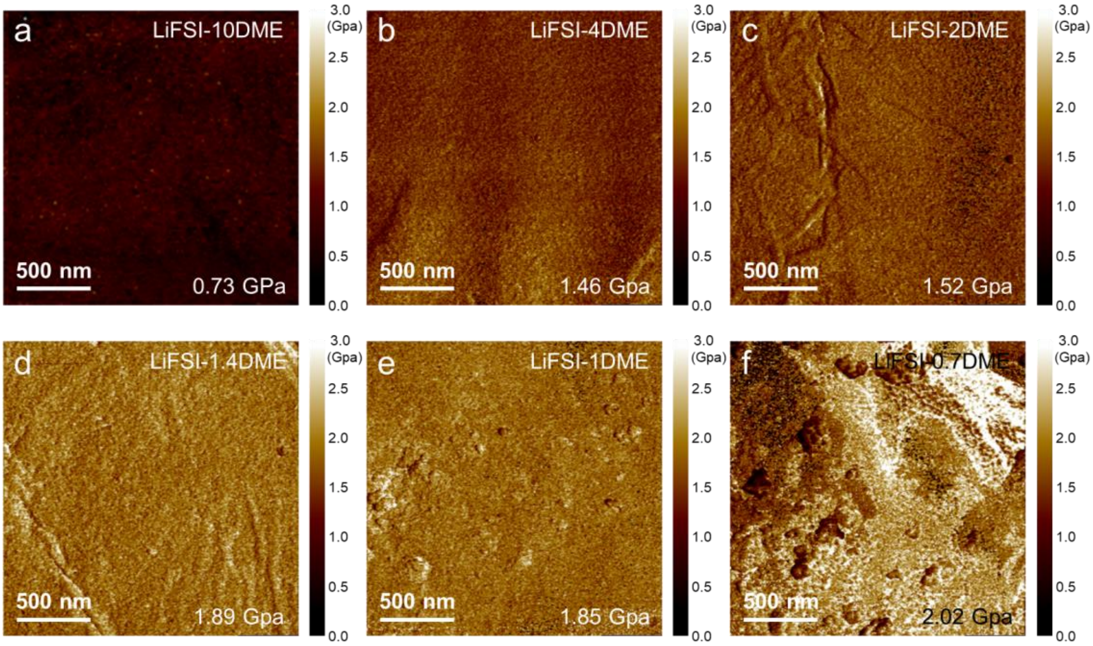

Supplementary Figure 18 | Modulus distributions of SEIs. (a-f) Peak force AFM quantitative nanomechanical mapping (QNM) images of 1 mAh cm^{-2} Li deposited on Cu foils in LiFSI-xDME electrolytes (x=10, 4, 2, 1.4, 1, 0.7, respectively). The current density is 0.5 mA cm^{-2}.

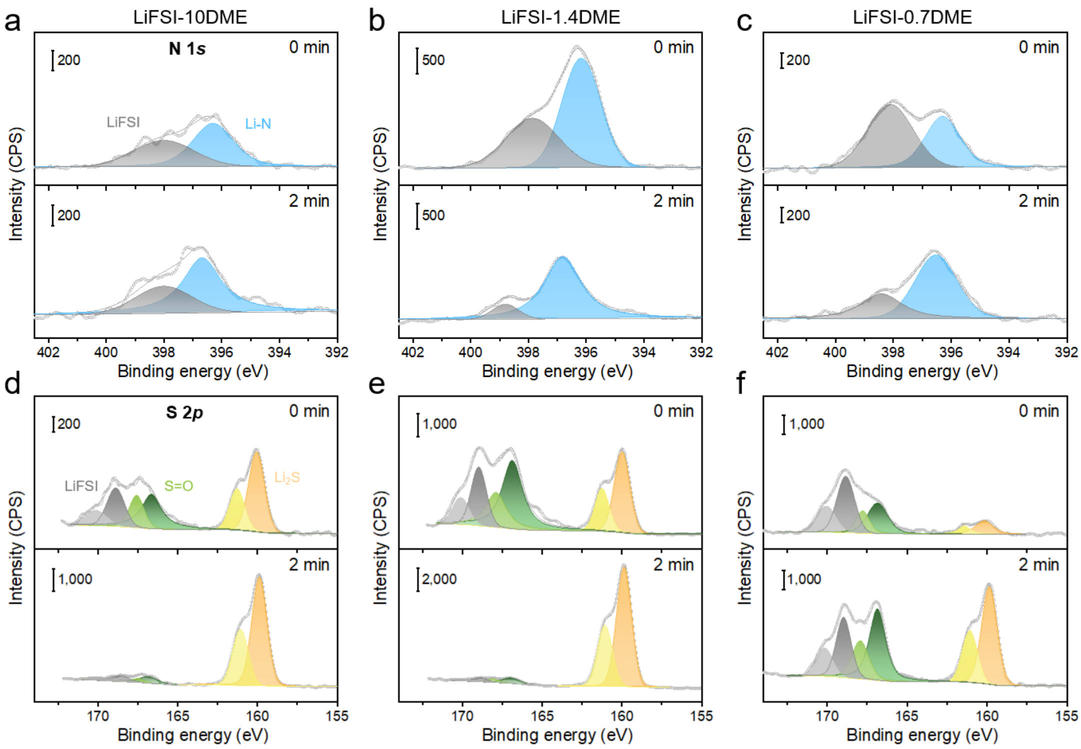

Supplementary Figure 19 | XPS N 1*s* and S 2*p* spectra of SEI. XPS (a, b, c) N 1*s* spectra and (d, e, f) S 2*p* spectra of SEI on deposited Li at different sputtering times (0 min and 2 min) in the (a, d) LiFSI-10DME, (b, e) LiFSI-1.4DME and (c, f) LiFSI-0.7DME electrolytes.

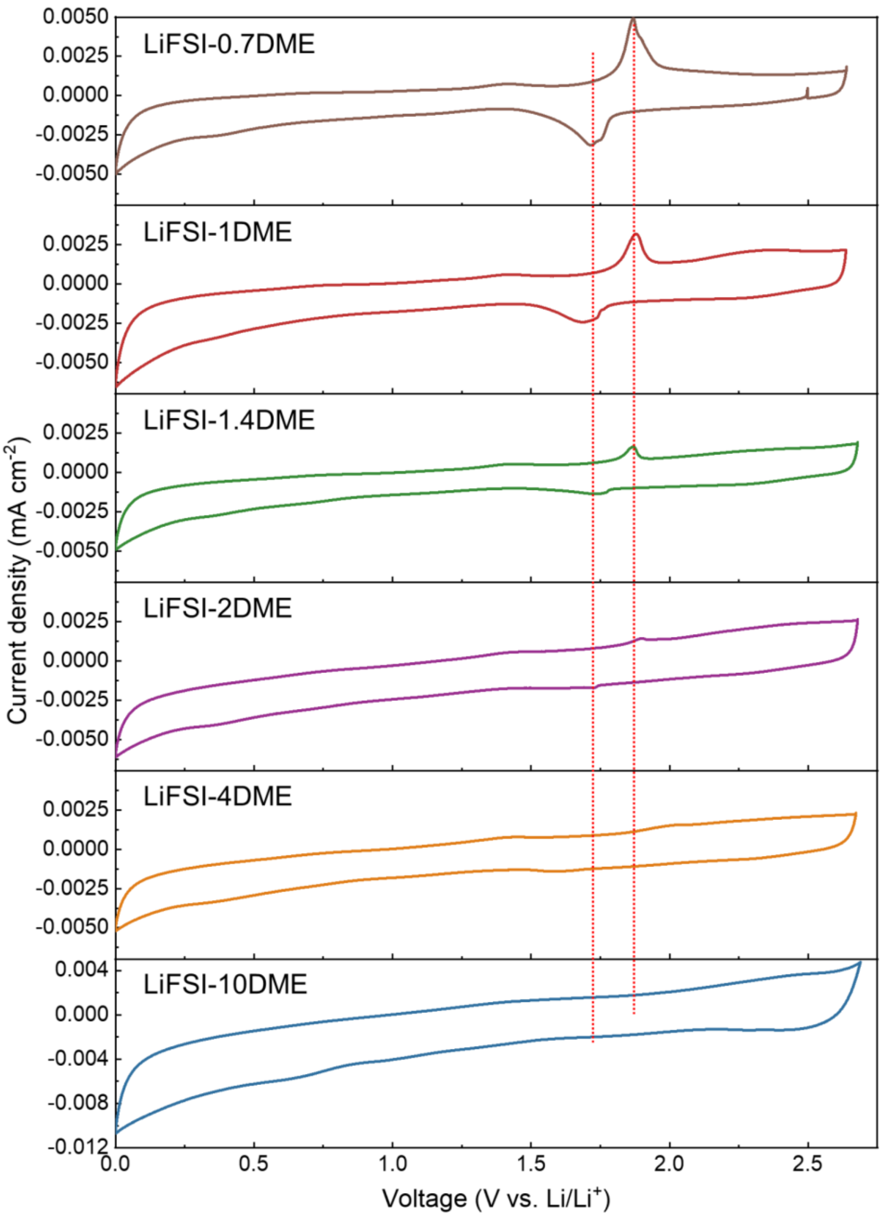

Supplementary Figure 20 | Electrochemical CV experiments. First cycle CV curves of Li/Cu half cells with different electrolytes over the voltage range from 2.7 V to 0 V.

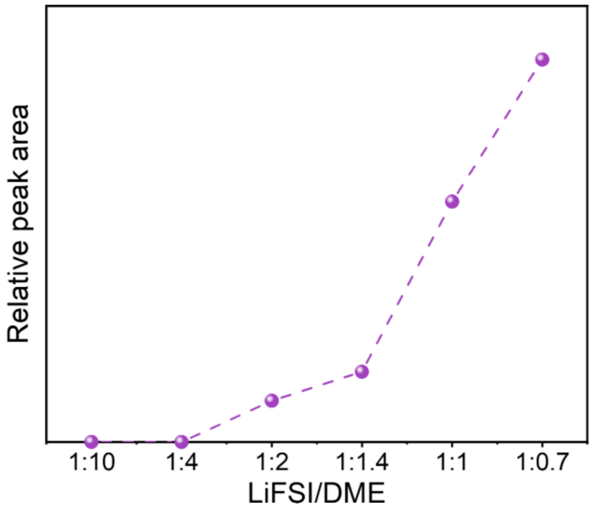

Supplementary Figure 21 | Reactivity comparison of FSI^- anions. Relative peak areas of the reduction peaks of the CV curves at ~1.7 V.
